# WebGIVI: a web-based gene enrichment analysis and visualization tool

**DOI:** 10.1186/s12859-017-1664-2

**Published:** 2017-05-04

**Authors:** Liang Sun, Yongnan Zhu, A. S. M. Ashique Mahmood, Catalina O. Tudor, Jia Ren, K. Vijay-Shanker, Jian Chen, Carl J. Schmidt

**Affiliations:** 1 Department of Animal and Food Sciences, University of Delaware, Newark, DE USA; 2 Department of Computer Science and Electrical Engineering, University of Maryland Baltimore County, 1000 Hilltop Circle, Baltimore, MD USA; 3 Department of Computer Science, Hangzhou Dianzi University, Hangzhou, 310018 Zhejiang Province People’s Republic of China; 4 Department of Computer and Information Sciences, University of Delaware, Newark, DE 19716 USA; 5 Center for Bioinformatics and Computational Biology, University of Delaware, Newark, DE 19711 USA; 6 Current address: Computing Service, The Samuel Roberts Noble Foundation, Ardmore, OK 73401 USA

**Keywords:** Visualization, eGIFT, Gene iTerm, Gene enrichment, Web development

## Abstract

**Background:**

A major challenge of high throughput transcriptome studies is presenting the data to researchers in an interpretable format. In many cases, the outputs of such studies are gene lists which are then examined for enriched biological concepts. One approach to help the researcher interpret large gene datasets is to associate genes and informative terms (iTerm) that are obtained from the biomedical literature using the eGIFT text-mining system. However, examining large lists of iTerm and gene pairs is a daunting task.

**Results:**

We have developed WebGIVI, an interactive web-based visualization tool (http://raven.anr.udel.edu/webgivi/) to explore gene:iTerm pairs. WebGIVI was built via Cytoscape and Data Driven Document JavaScript libraries and can be used to relate genes to iTerms and then visualize gene and iTerm pairs. WebGIVI can accept a gene list that is used to retrieve the gene symbols and corresponding iTerm list. This list can be submitted to visualize the gene iTerm pairs using two distinct methods: a Concept Map or a Cytoscape Network Map. In addition, WebGIVI also supports uploading and visualization of any two-column tab separated data.

**Conclusions:**

WebGIVI provides an interactive and integrated network graph of gene and iTerms that allows filtering, sorting, and grouping, which can aid biologists in developing hypothesis based on the input gene lists. In addition, WebGIVI can visualize hundreds of nodes and generate a high-resolution image that is important for most of research publications. The source code can be freely downloaded at https://github.com/sunliang3361/WebGIVI. The WebGIVI tutorial is available at http://raven.anr.udel.edu/webgivi/tutorial.php.

**Electronic supplementary material:**

The online version of this article (doi:10.1186/s12859-017-1664-2) contains supplementary material, which is available to authorized users.

## Background

High-throughput technologies provide biologists with large lists of genes or proteins when they compare expression data between two biological states (e.g., normal tissue vs. cancer tissue). Grouping enriched genes to known biological processes and pathways is a common strategy for understanding the biology that underlies the differences between the two states. Approaches include GO enrichment analysis such as DAVID [1, 2], GOEAST [3] and Gorilla [4], and pathway analysis such as KEGG [5] and Reactome [6].

### eGIFT

eGIFT [7] uses a text-mining method to identify informative terms (iTerms) for individual genes. iTerms are not limited to gene ontology (GO) terms; they also capture more detailed biological knowledge. Consequently, eGIFT provides a finer grained interpretation of gene lists than GO analysis. The current gene analysis results of eGIFT provide users with a list of ranked iTerms and their associated genes in a tabular format. A graphic representation of these gene and iTerm relations would allow biologists to better interpret their input gene lists or gene-iTerm pair lists. This often captures the biological concept enriched in the input data.

### Visualization tool

An effective visualization of large data sets can provide biologists with means to discover buried relationships in complex data sets. Currently, several different visualization tools are used to capture the relationship between genes, protein and networks, such as Arena 3D [8], Medusa [9], Ondex [10], Osprey [11], Pajek [12], BioLayout Express^3D^ [13], Cytoscape [14] and ProViz [15]. However, most of these tools need to be installed on a local computer and require plugins or third party software such as Java Runtime Environment. Installation and maintenance of such tools can be difficult for those unfamiliar with computer system administration. In contrast, web-based tools offer the ability to visualize relationships without the overhead of having access to system administrators. Cytoscape.js [16] and D3.js [17] are the most popular visualization JavaScript libraries that can be applied to visualize network and biomolecule interactions. There are several successful web based visualization tools that use the Cytoscape and D3 JavaScript libraries in a biological context. For example, BNVC [18], a web-based visualization tool of biomolecular networks, can be used to compare two similar networks. PINV [19], also a web-based protein-protein interaction network visualizer, provides complex interaction networks with the ability to query, filter and group data of interest. However, complex graphs rendered by such tools are difficult to interpret when analyzing data with hundreds of nodes and edges. WebGIVI addresses the issue of visualizing large data sets by adopting a concept map method for visualization. The concept map aligns nodes on different layers and automatically calculates the distance between layers. This maximizes the amount of information that can be displayed despite limited physical size of the screen. In addition, a zoom-in feature of Concept Map allows the user to scale the graph for a better view. Now, a user can analyze hundreds of nodes in a high-resolution image produced by WebGIVI that can be saved and is suitable for publication.

Interactive WebGIVI provides an integrated graph to help users generate biological hypotheses. A database of rate-limiting genes, identified by text mining and manually verified, was also integrated into WebGIVI. All genes encoding rate-limiting products are colored in the visualization. Expression data that is uploaded to WebGIVI can also be color-coded according to expression levels in two different biological states. These features are accomplished by using PHP, Cytoscape.js and D3.js to generate a powerful and interactive web based visualization tool that implements gene enrichment analysis and gene and iTerms visualization.

## Implementation

**Fig. 1:**
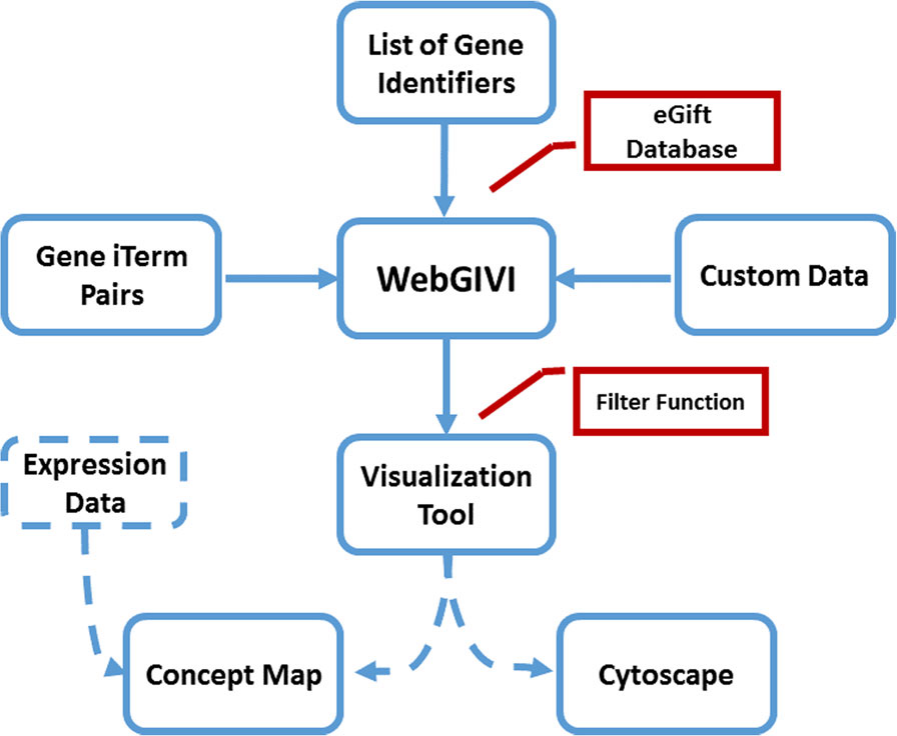
Flow chart of WebGIVI tool. Gene iTerm Pair indicates gene symbol and its associated informative term

The flow chart of the data processing is depicted in Fig. 1. Currently, WebGIVI accepts multiple input data formats including: NCBI Entrez gene ID, UniProt [20], or Ensemble [21] Gene IDs. Prior to accessing WebGIVI, the user will have identified genes of interest in their biological system. The WebGIVI input list is used to retrieve the gene symbols and iTerms through an eGIFT API. In the retrieved list of gene symbol-iTerm pairs, the first column contains the gene symbols, and the second column contains the gene-associated iTerms. Interactive WebGIVI also supports uploading and visualization of any two-column tab separated data. For instance, gene symbols and related pathways data can be visualized.

WebGIVI has three major functions:

1. *Visualization*. Interactive table visualization includes sorting and deleting functions that help users reduce the graph to only iTerms and genes of interest. GUI functions in the Concept Map visualization include switching, filtering, sorting, searching, grouping and saving the data in tab delimited text files or as high-resolution images.
2. *Integration of Rate-limiting and Expression Data*. Rate limiting gene products determine the flow rate of metabolites or signal through pathways. We have identified 150 rate limiting gene products via text-mining of primary literature and biochemistry textbooks. This list was manually verified (CJS), and integrated into WebGIVI.
  When a user enters an expression data list, genes that encode a rate limiting gene product are colored when visualizing gene:iTerm pair in the concept map. If such genes are differentially regulated they are likely have a significant impact on the overall rate of metabolite or signal flow through their respective pathways. Furthermore, to emphasize certain features of their data, users can upload expression files that highlight nodes that they consider informative.

3. *Link-out Functions*. Nodes and edges provide link outs to gene specific, iTerms or gene:iTerm pairs via the NCBI, UniPROT, or eGIFT database. For example, gene-iTerm pairs link to eGIFT sentence web page, which contain sentences with the gene and iTerm highlighted in the text.

## Results and discussion

### Data filtering functions

*Sorting Functions*: eGIFT uses a precomputed text-mining database that has extracted all gene associated informative terms (iTerm) from PubMed abstracts. After submitting a gene list to WebGIVI, a table is returned to the user containing the iTerms associated with the input genes. By hovering over an iTerm, the user can see the genes associated with that iTerm. The default list is sorted based on the Fisher’s exact test p-value, but the user can choose to sort based on alphabetical order, the gene ontology group (process, function, compartment or unclassified) to which the iTerm has been classified or the frequency of appearance of each iTerm.

*Editing Functions*: Not all iTerms are informative in all use cases, but could be important to others. For example, ‘in situ hybridization’ is an irrelevant iTerm to our use case scenario but will be interesting to researchers who might want to apply this experimental method to their own work. However, some iTerms are highly likely to be non-informative. To remove such iTerms a “blacklist” has been developed that includes terms such as “some cell” or “10 fold” that are typically non-informative to the general WebGIVI user. Since the developers of WebGIVI cannot be certain that a given iTerm is irrelevant to all users, the returned iTerm list includes the blacklisted terms; a checkbox is provided that allows the user to hide any terms that are included in the blacklist. It is also beneficial to the user to also be able to filter out irrelevant iTerms in the context of their study, and only save iTerms of direct relevance. Once data is submitted on WebGIVI’s homepage, the returned list will allow the user to delete iTerms from the results table using deleting functions. If the user prefers, they can choose not to prefilter but visualize data in Concept Map or Cytoscape directly.

### A biological use case scenario

**Fig. 2:**
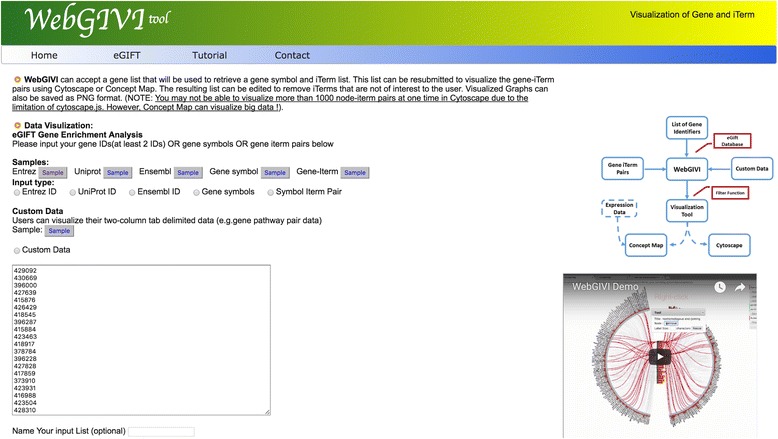
Submission interface for WebGIVI. Gene lists can be input in several different formats and named in the text field above the submit button. To the right of the page is a short video demonstrating various WebGIVI functionalities

**Fig. 3:**
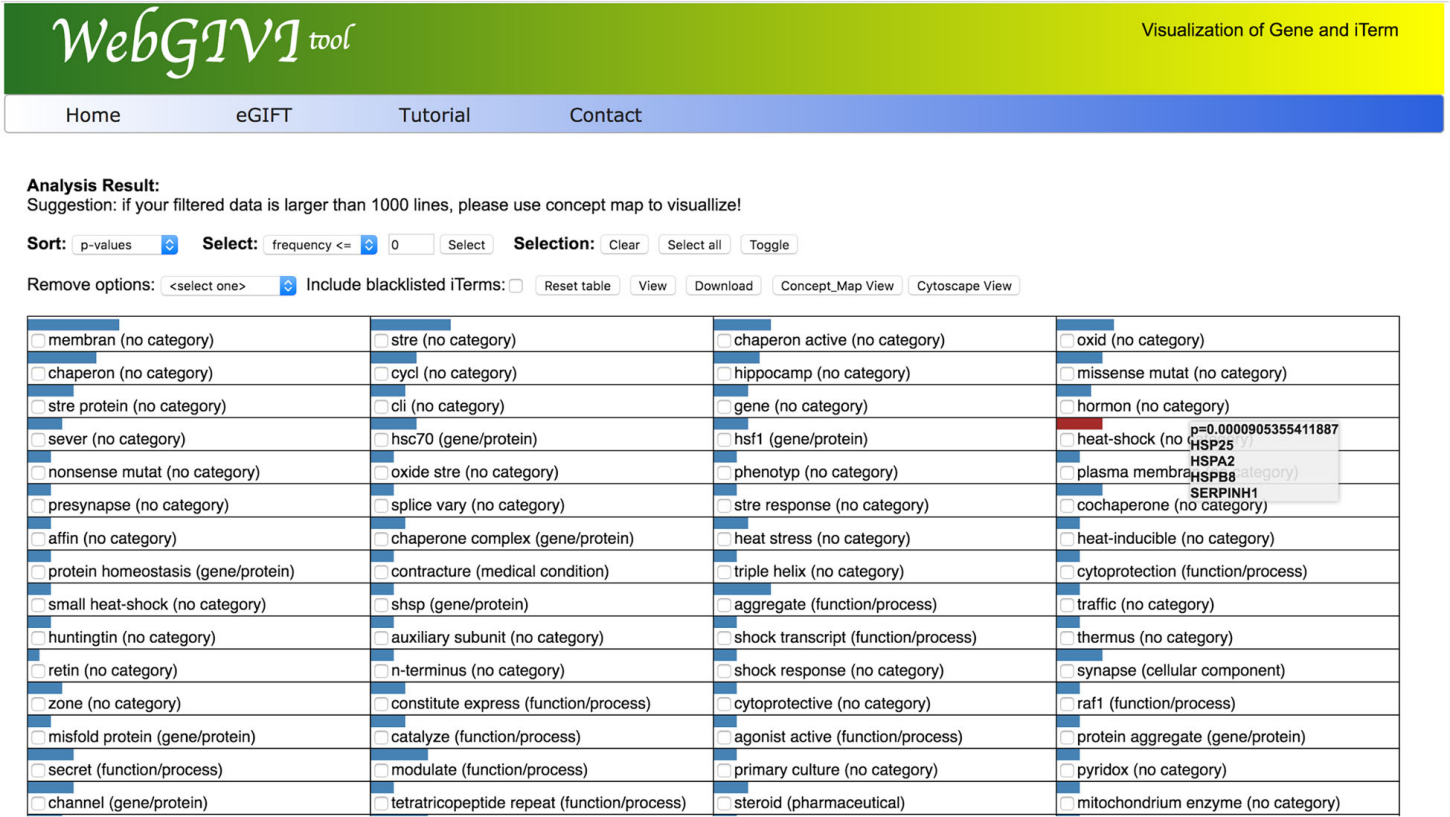
Results pages containing iTerms enriched in the input gene list. In this case the list was sorted by *p*-values as determined by Fisher’s exact test. One can use the Frequency text box to eliminate iTerms that have fewer genes associated with them based on the user’s cutoff. The Selection buttons allow one to “Select All”, Clear the selections or Toggle selected iTerms. Also, individual iTerms can be selected by clicking. The selection features are used to remove irrelevant iTerms based on the users knowledge. One can include blacklisted iTerms (see text) by selecting the check box. The Reset Table button can be used to recreate the starting table should errors be made while deleting iTerms thought to be irrelevant. The Concept Map View and Cytoscape View open new windows with the corresponding representations of the iTerm:gene pairs. Hovering over an iTerm box will display the genes associated with that iTerm from the input list along with the *p*-value of that iTerm

**Fig. 4:**
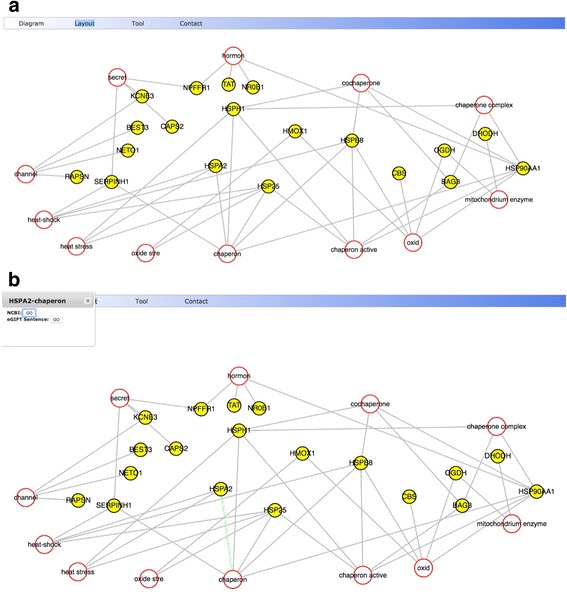
**a** Cytoscape View of iTerm:gene pairs. **b** *Right clicking* on an edge connecting two nodes will activate a pop up window allowing the user to connect to either NCBI or eGIFT sentences (see text)

**Fig. 5:**
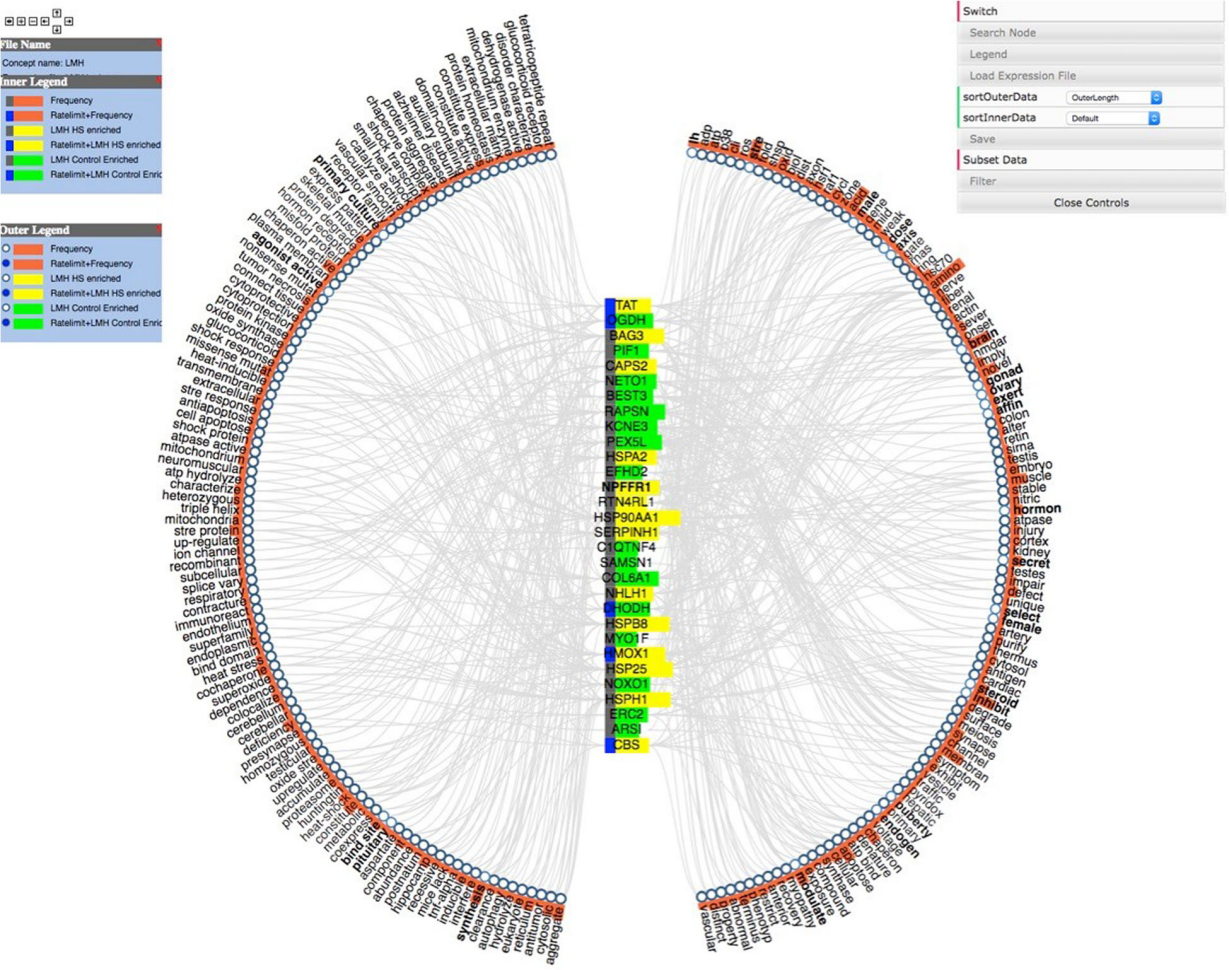
Concept Map view of iTerm:gene pairs. Genes from the input list with iTerms are in the vertical column at the center and iTerms are around the outside. The panel to the left is the legend explaining the graph, while the panel to the *right* allows manipulation of the output. This allows the user to switch the positions of the genes and iTerms, Search for specific genes or iTerms and to toggle the legend window on or off. The Load Expression File allows the user to load a file that indicates if a gene was up or down regulated in the experiment. *Yellow* corresponds to up while *green* to down regulated genes. The Save button allows the user to save either the graph text as a tab delimited two-column files or save the image. The Filter button allows the user to choose the minimum number of edges that a node must have to be visualized. See Fig. 6 for explanation of Subset Data

We used the Sun et al. white-leghorn hepatocellular (LMH) cell heat stress dataset [22], which is a RNA-Seq study of LMH cells under heat stress. This study identified a total of 235 up-regulated and 578 down-regulated genes. Figure 2 shows a completed WebGIVI submission page with a portion of the regulated genes from the LMH study (Additional file 1). In this case we used Entrez gene identification numbers. Following submission, an iTerm list (Fig. 3) is returned that can be sorted alphabetically, by frequency, Gene Ontology categories, or by p-value as determined by the Fisher’s exact test. In this case the list is sorted by p-value. Hovering over an iTerm will show the corresponding p-value, along with the genes from the list associated with that iTerm. One can choose to display iTerms that have been blacklisted by checking the “Include blacklisted items”. You can also select irrelevant iTerms by right clicking and delete them using the remove options. Users can view the output in either Cytoscape (Fig. 4) or as a Concept Map (Fig. 5) by selecting the appropriate buttons. The default mode in Cytoscape generates a force graph (Fig. 4a) and clicking on an edge connecting a gene product to an iTerm pops up a window that allows the user to connect to either NCBI or eGIFT (Fig. 4b). Additional view modes include tree or circle that are accessible by the Layout button.

While Cytoscape can be useful for small graphs, the Concept map view is generally easier to use for larger input data sets. In the default view, gene symbols are displayed in a column at the center of the view, while iTerms are displayed as a wheel around the gene symbols (Fig. 5). If necessary, additional layers are automatically added to display more gene:iTerm relations. Several attributes are visible in the Concept Map view. In this case we have uploaded a file indicating how the gene was regulated by heat stress using the Load Expression File button. Genes highlighted in yellow are enriched under heat stress while those in green are enriched in the control (thermoneutral) samples. In addition, genes encoding rate limiting gene products are indicated by a blue rectangle added to the symbol.

**Fig. 6:**
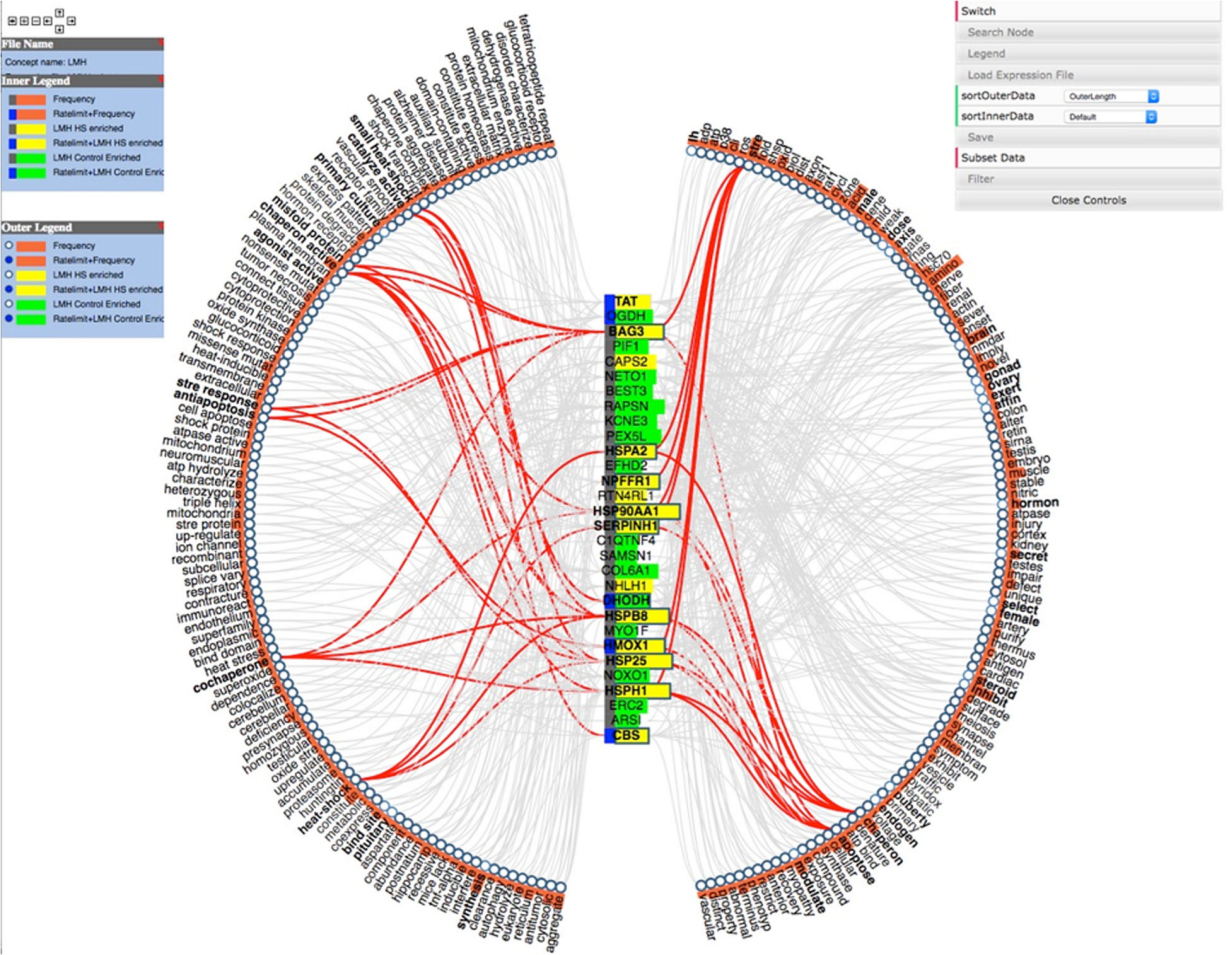
Clicking on either iTerm or gene nodes will activate connecting edges. Pushing the Subset Data button will create a new graph (Fig. 7) containing only those selected nodes

**Fig. 7:**
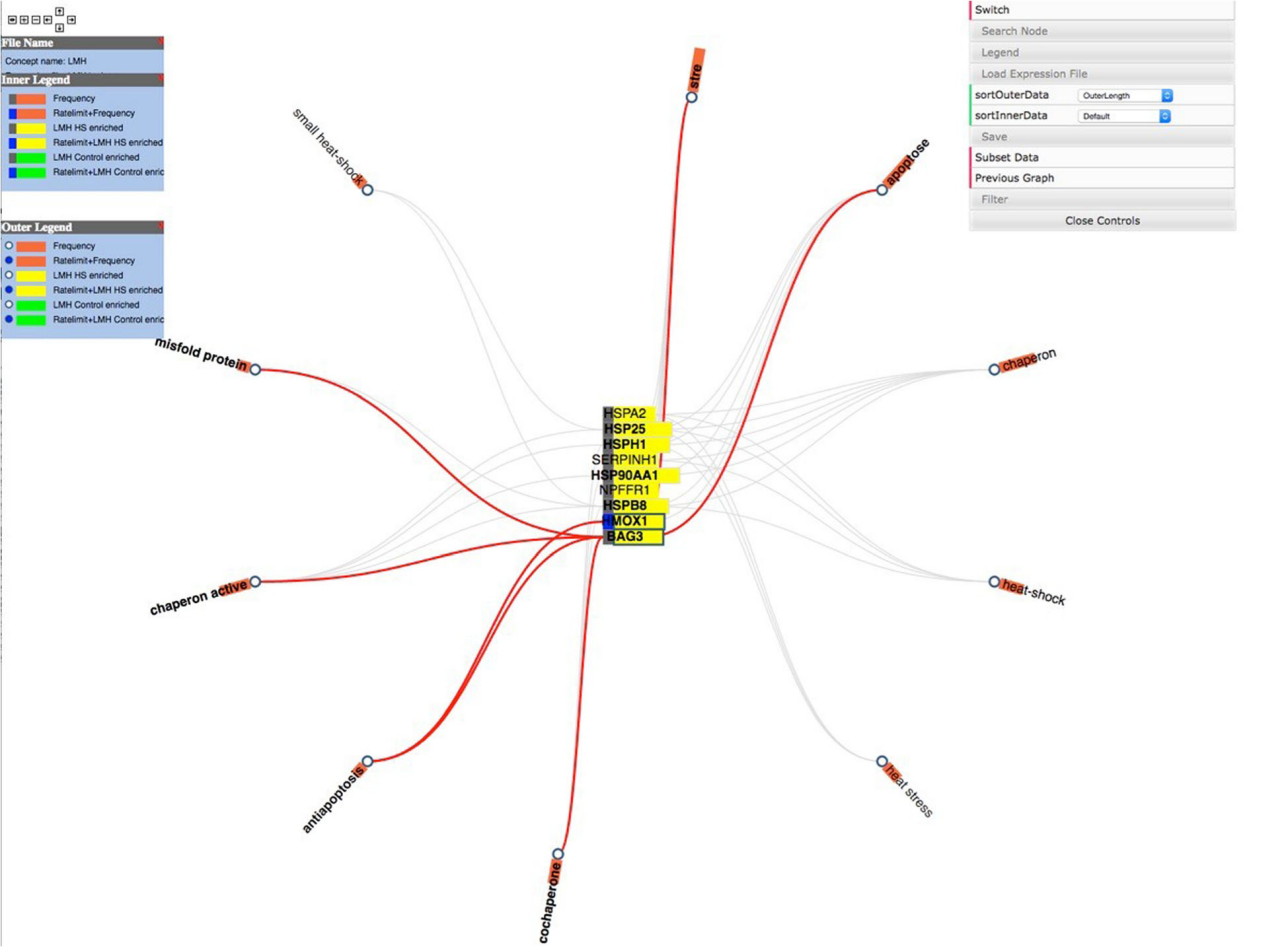
Subgraph created from selections indicated in Fig. 6

**Fig. 8:**
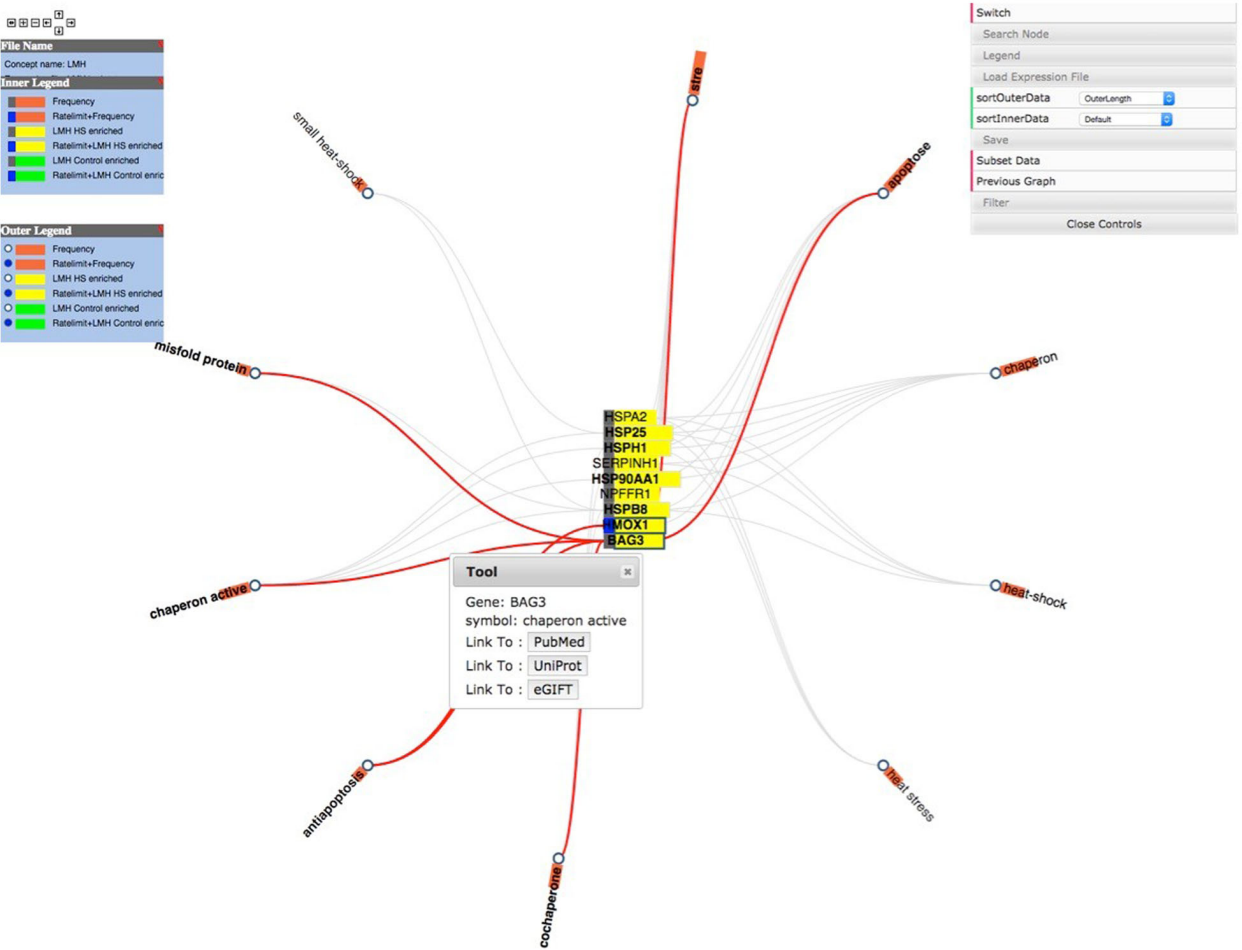
Link outs to other resource. *Right clicking* on an active edge opens a pop up window allow the user to click out to PubMed, UniProt or eGIFT resources

**Fig. 9:**
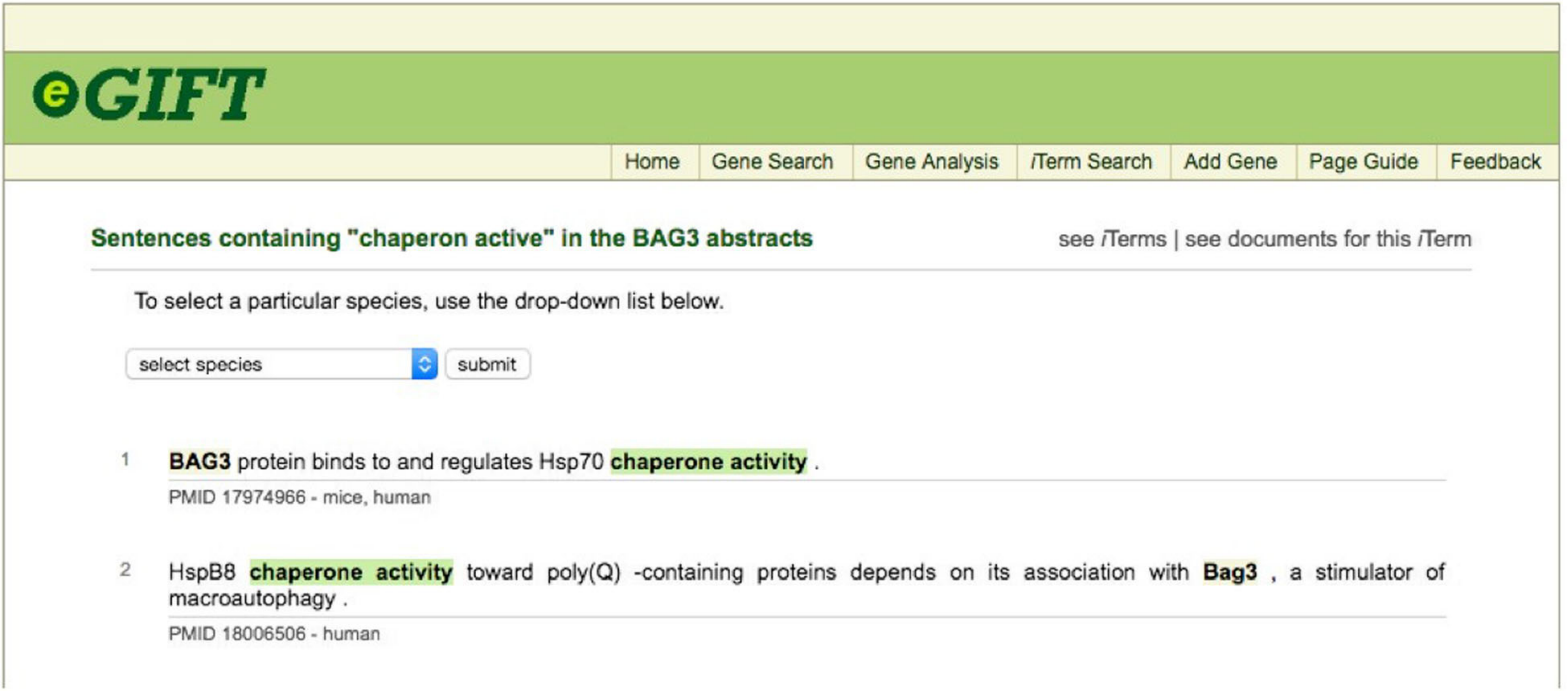
Sentences displayed by WebGIVI. Sentences containing the gene BAG3 and the corresponding iTerm “Chaperone Activity” that can be accessed by clicking on the appropriate edge (see Fig. 9)

In the concept map view users can right click to select either genes or iTerms. Selecting one will create a gene:iTerm edge (Fig. 6) then clicking on the Subset Data button will create a new concept map with just the selected gene and iTerms (Fig. 7). This is useful to allow an investigator to link genes with similar iTerms for subsequent investigation. At any point, right clicking on an active edge will open a window that can be used to connect to PubMed, UniProt or eGIFT (Fig. 8). Linking out to NCBI database will search that database with the gene and iTerm, and retrieve links to abstracts that contain those two search terms. For example when searching for a gene:iTerm pair such as HSP90AA1 and the iTerm “chaperone” the search will be in the syntax “HSP90AA1 AND chaperone” and the results will include all abstracts that include both the HSP90AA1 and chaperone. Linking to UniProt will access a search page allowing the user to view the UniProt entry for the gene product. Linking to eGIFT web pages provides users with the sentences extracted from the literature that contain the gene and iTerm pair (Fig. 9). This feature of WebGIVI greatly facilitates the user’s understanding of the gene product’s function. In addition, the PMID under a sentence links to the PubMed abstract pages from which the sentence was extracted. This further aids in placing the gene product in biological context. The ability to provide users with these sentences is a unique facet of WebGIVI functionality.

Interactive WebGIVI also provides a way to save the current view: users can export all data on the Concept Map as a two-column tab separated file, which can be resubmitted in WebGIVI to obtain the same graph. This feature allows a user to readily share the data with collaborators. Users can also export the graph as a scalable vector graphics (SVG) format, which can be transformed to high resolution image types via readily accessible image conversion web sites.

### Comparison of WebGIVI, DAVID and AmiGO2 analysis

Incorporating the text-mining tool, WebGIVI, into the analysis of high-throughput transcriptome experiments complements Functional Annotation clustering provided by DAVID and GO analysis provided by AmiGO2 [23]. DAVID is an online knowledgebase that can output a list of enriched biological concepts from an input list of gene identifiers. The site makes use of multiple resources including the Gene Ontology, the KEGG pathway database, Interpro [24], along with several others. Amigo2 is a product of the Gene Ontology consortium and allows users to submit gene lists and identify enriched GO terms. We chose to compare WebGIVI with DAVID and Amigo2 because these resources are easy to use and have been widely adopted by the scientific community.

Heat stress has been implicated in affecting cell cycle regulatory processes including DNA synthesis, DNA repair, cell cycle checkpoints, cell proliferation, and spindle formation. The objective was to compare the ability of DAVID, AmiGO2 and WebGIVI to identify genes affecting cell cycle regulation that are up-regulated by heat stress in the liver of chickens (Jastrebski et al. manuscript submitted). Genes whose expression was increased by heat stress were identified and submitted to Amigo2, DAVID, and WebGIVI for comparative analysis. Genes recognized by each analytical method as associated with the concept of cell cycle regulation (including cell cycle, DNA replication, DNA repair, checkpoint) were considered in this comparison of methods (see Additional file 2 for complete list of identified genes).

**Fig. 10:**
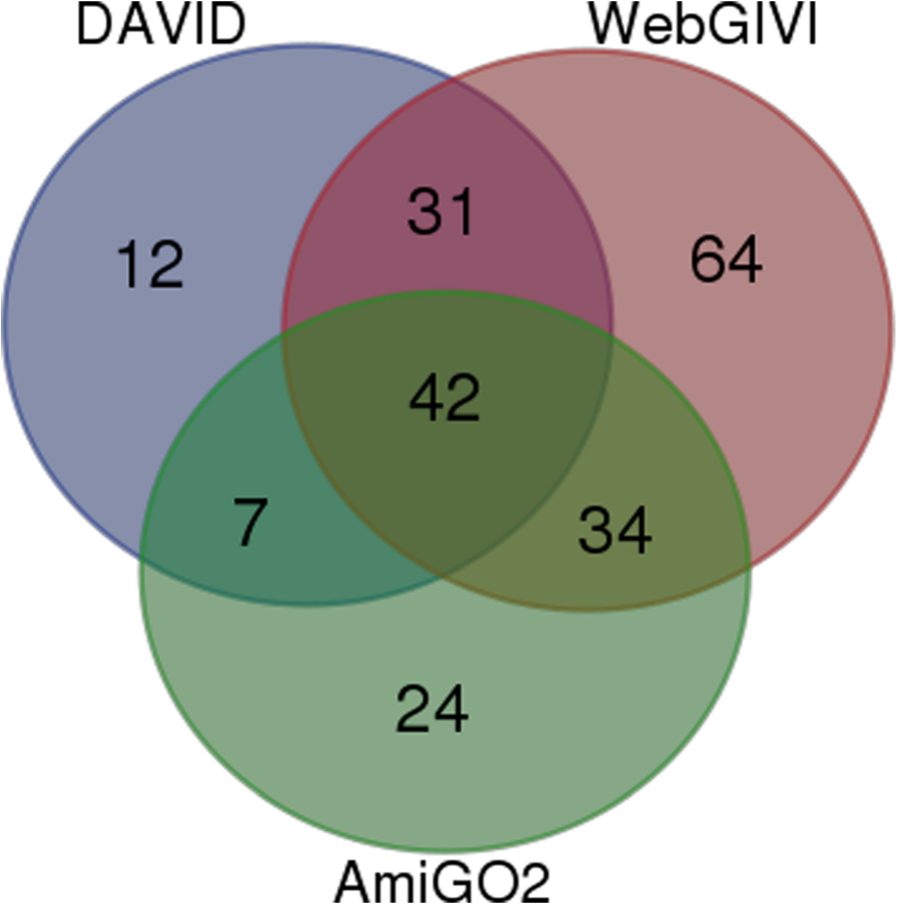
Venn diagram depicting the number of genes associated with the biological concept of “cell cycle regulation” (see text) by DAVID, AmiGO2 and WebGIVI

In combination DAVID, AMIGO2 and WebGIVI identified a total of 214 genes affecting cell cycle regulation as enriched by heat stress in the liver. WebGIVI identified the largest percentage of total genes (80%) and uniquely identified the greatest percentage of genes (30%) not recognized by either DAVID or AMIGO2 (Table 1). However, WebGIVI missed 7 genes that were captured by either DAVID or Amigo2 (Fig. 10). Taken together, this analysis indicates that multiple approaches are best for categorizing the biology embodied in gene lists, and that WebGIVI provides an important contribution to these analyses.

**Table 1:** Comparative analysis of WebGIVI, DAVID and AmiGO2

Analysis tool	Percent of total	Percent unique
WebGIVI	80%	30%
DAVID	42%	6%
AmiGO2	50%	11%

The three tools identified a total of 214 genes associated with the biological concept of cell cycle regulation” (see text). The numbers indicate the percentage of the 214 genes identified by the different tools along with the number of genes uniquely identified by the corresponding tools. In this analysis, WebGIVI identified 64 (30%) genes as associated with cell cycle regulation that were not associated with this concept by either DAVID or AmiGO2 (see Fig. 10)

### Future work

WebGIVI can be used to visualize not only eGIFT data, but also any two-column relationship data. For example, microRNA and target genes, kinase and substrate pairs, and protein-protein interactions could all be visualized with WebGIVI. Our tool can also be used as an extension for other tools. Compared to other visualization tools, our web-based gene iTerm visualization tool is highly customizable. Users can easily upload their own data and edit their data in the graph. No pre-installed or third party software such as Java Runtime Environment is required to visualize users’ data. While we are developing a WebGIVI blacklist of iTerms we believe are not informative, users still need to examine all iTerms and remove ones they find uninformative manually. This inability of WebGIVI to learn as user’s preferences and automatically remove iTerm provides room for future improvement. Potentially, machine-learning techniques could be applied to remove such iTerms. Future work will also integrate more bioinformatics databases such as protein kinase database and transcriptional factor database to enable the user to discover more interesting biological relationships and improve the usability of WebGIVI.

## Conclusions

Interactive WebGIVI tool provides an integrated visualization and gene enrichment analysis tool. It helps biologists to visualize genes and iTerms online, makes sense of their biological data, and is useful to generating biological hypotheses from high throughput data.

## Availability and requirements

Project name: WebGIVI: Web-based Gene and Iterm Visualization Tool

Project home page: http://raven.anr.udel.edu/webgivi/

Source code: https://github.com/sunliang3361/WebGIVI

Operation system(s): Web based, Platform independent

Programming language: HTML, CSS, JavaScript, PHP

Other requirements: Modern Browser

License: BSD License

Any restrictions to use by non-academics: None.

## Additional files

Additional file 1: NCBI Gene Entrez ID List for the Case Scenario. This file contains NCBI gene Entrez ID list. The first column is Entrez ID, and the second column is the gene symbol. (XLSX 9 kb)

Additional file 2: NCBI Entrez IDs for Comparison of WebGIVI, DAVID and AmiGO2. This file contains NCBI gene Entrez ID list which is identifed by WebGIVI, DAVID and AmiGO2 to be associated with the concept of cell cycle regulation. (TXT 1 kb)

## Abbreviations

GO: Gene ontology
iTerm: Informative term
LMH: White-leghorn hepatocellular cell
SVG: Scalable vector graphics
